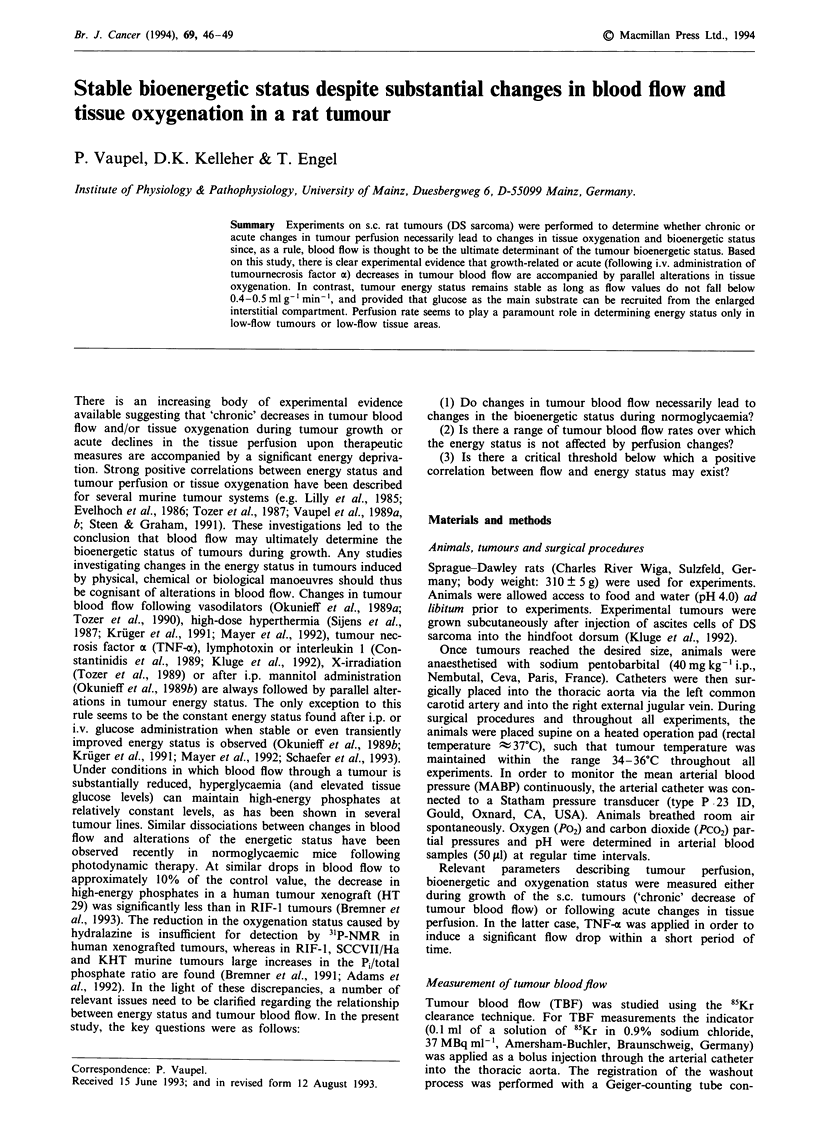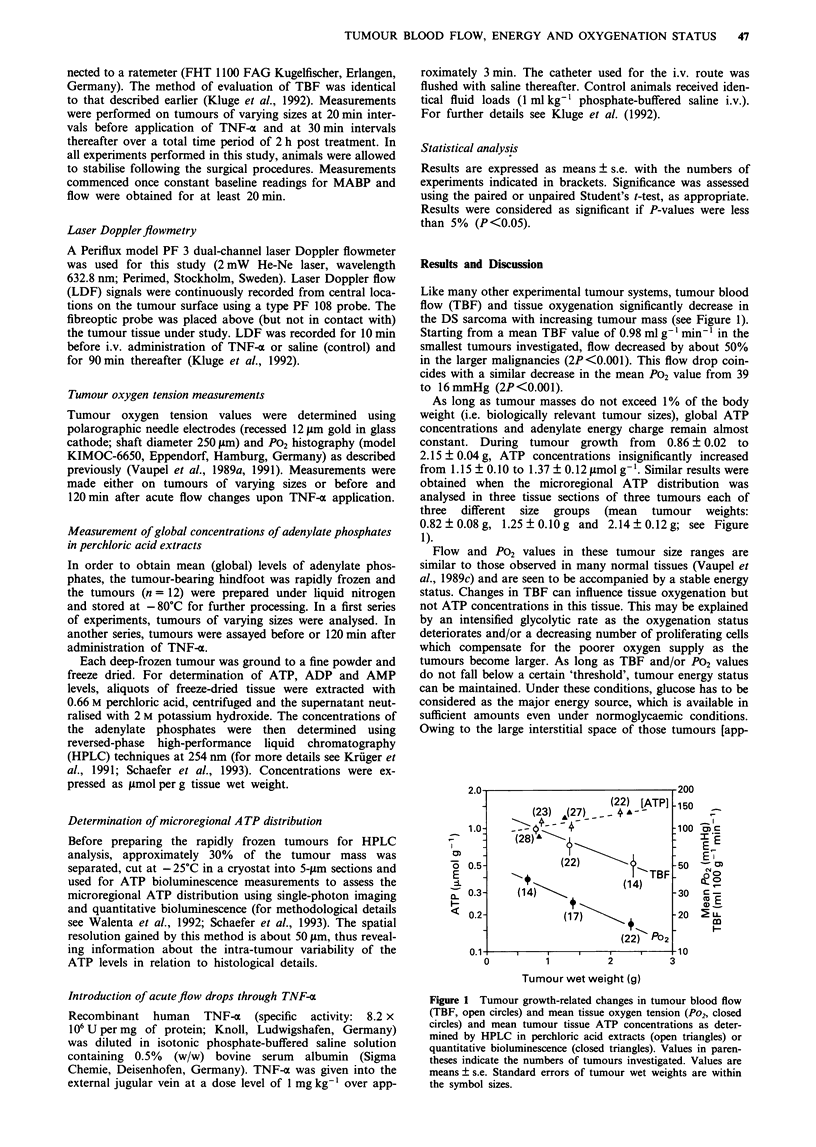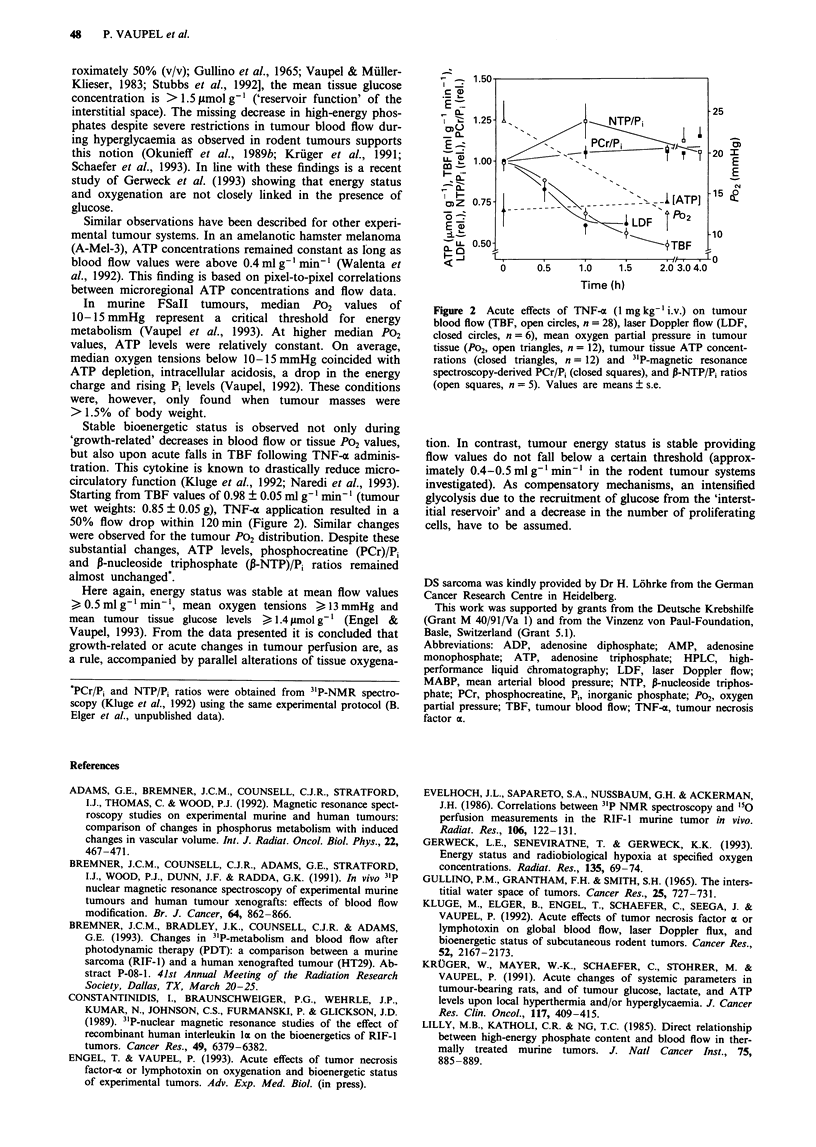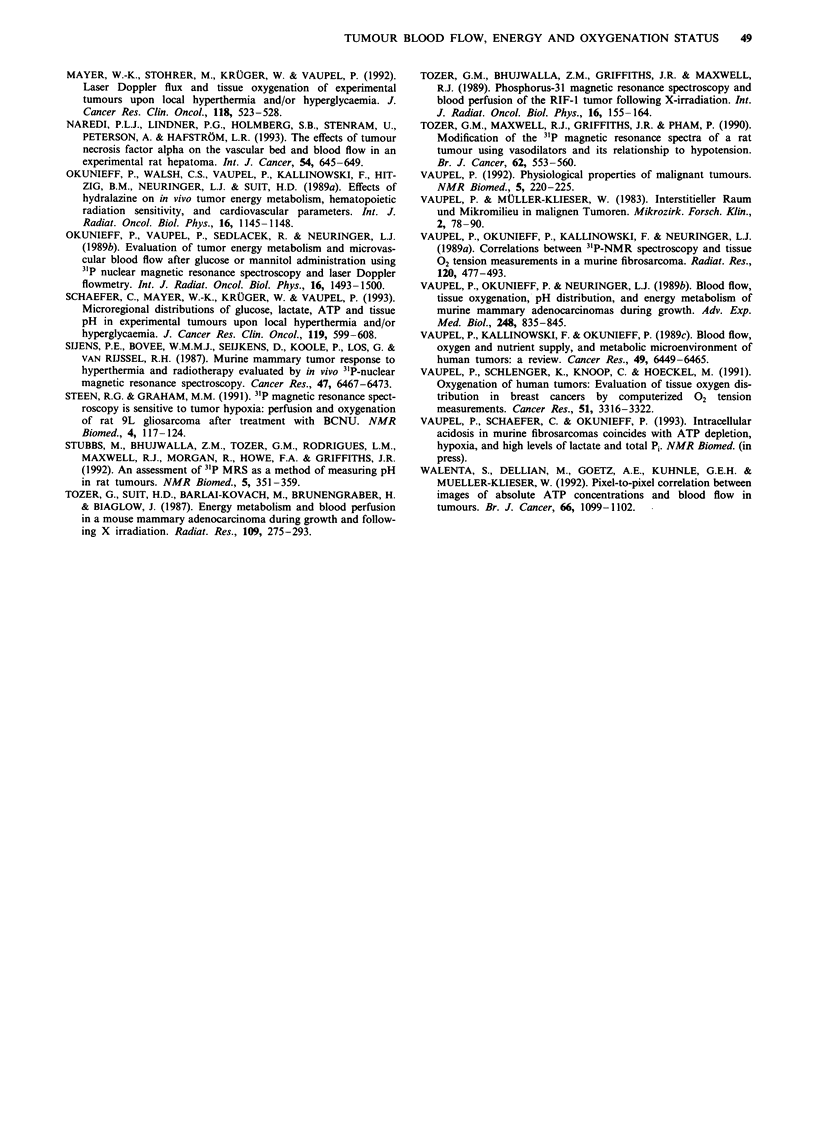# Stable bioenergetic status despite substantial changes in blood flow and tissue oxygenation in a rat tumour.

**DOI:** 10.1038/bjc.1994.7

**Published:** 1994-01

**Authors:** P. Vaupel, D. K. Kelleher, T. Engel

**Affiliations:** Institute of Physiology & Pathophysiology, University of Mainz, Germany.

## Abstract

Experiments on s.c. rat tumours (DS sarcoma) were performed to determine whether chronic or acute changes in tumour perfusion necessarily lead to changes in tissue oxygenation and bioenergetic status since, as a rule, blood flow is thought to be the ultimate determinant of the tumour bioenergetic status. Based on this study, there is clear experimental evidence that growth-related or acute (following i.v. administration of tumour necrosis factor alpha) decreases in tumour blood flow are accompanied by parallel alterations in tissue oxygenation. In contrast, tumour energy status remains stable as long as flow values do not fall below 0.4-0.5 ml g-1 min-1, and provided that glucose as the main substrate can be recruited from the enlarged interstitial compartment. Perfusion rate seems to play a paramount role in determining energy status only in low-flow tumours or low-flow tissue areas.


Br. J. Cancer (1994), 69, 46-49 © Macmillan Press Ltd., 1994

### Stable bioenergetic status despite substantial changes in blood flow and tissue oxygenation in a rat tumour

P. Vaupel, D.K. Kelleher & T. Engel

*Institute of Physiology & Pathophysiology, University of Mainz, Duesbergweg 6, D-55099 Mainz, Germany.*

**Summary** Experiments on s.c. rat tumours (DS sarcoma) were performed to determine whether chronic or acute changes in tumour perfusion necessarily lead to changes in tissue oxygenation and bioenergetic status since, as a rule, blood flow is thought to be the ultimate determinant of the tumour bioenergetic status. Based on this study, there is clear experimental evidence that growth-related or acute (following i.v. administration of tumournecrosis factor α) decreases in tumour blood flow are accompanied by parallel alterations in tissue oxygenation. In contrast, tumour energy status remains stable as long as flow values do not fall below 0.4–0.5 ml $g^{-1}$ $min^{-1}$, and provided that glucose as the main substrate can be recruited from the enlarged interstitial compartment. Perfusion rate seems to play a paramount role in determining energy status only in low-flow tumours or low-flow tissue areas.

There is an increasing body of experimental evidence available suggesting that 'chronic' decreases in tumour blood flow and/or tissue oxygenation during tumour growth or acute declines in the tissue perfusion upon therapeutic measures are accompanied by a significant energy deprivation. Strong positive correlations between energy status and tumour perfusion or tissue oxygenation have been described for several murine tumour systems (e.g. Lilly *et al.*, 1985; Evelhoch *et al.*, 1986; Tozer *et al.*, 1987; Vaupel *et al.*, 1989*a*, *b*; Steen & Graham, 1991). These investigations led to the conclusion that blood flow may ultimately determine the bioenergetic status of tumours during growth. Any studies investigating changes in the energy status in tumours induced by physical, chemical or biological manoeuvres should thus be cognisant of alterations in blood flow. Changes in tumour blood flow following vasodilators (Okunieff *et al.*, 1989*a*; Tozer *et al.*, 1990), high-dose hyperthermia (Sijens *et al.*, 1987; Krüger *et al.*, 1991; Mayer *et al.*, 1992), tumour necrosis factor α (TNF-α), lymphotoxin or interleukin 1 (Constantinidis *et al.*, 1989; Kluge *et al.*, 1992), X-irradiation (Tozer *et al.*, 1989) or after i.p. mannitol administration (Okunieff *et al.*, 1989*b*) are always followed by parallel alterations in tumour energy status. The only exception to this rule seems to be the constant energy status found after i.p. or i.v. glucose administration when stable or even transiently improved energy status is observed (Okunieff *et al.*, 1989*b*; Krüger *et al.*, 1991; Mayer *et al.*, 1992; Schaefer *et al.*, 1993). Under conditions in which blood flow through a tumour is substantially reduced, hyperglycaemia (and elevated tissue glucose levels) can maintain high-energy phosphates at relatively constant levels, as has been shown in several tumour lines. Similar dissociations between changes in blood flow and alterations of the energetic status have been observed recently in normoglycaemic mice following photodynamic therapy. At similar drops in blood flow to approximately 10% of the control value, the decrease in high-energy phosphates in a human tumour xenograft (HT 29) was significantly less than in RIF-1 tumours (Bremner *et al.*, 1993). The reduction in the oxygenation status caused by hydralazine is insufficient for detection by $^{31}$P-NMR in human xenografted tumours, whereas in RIF-1, SCCVII/Ha and KHT murine tumours large increases in the $P_i$/total phosphate ratio are found (Bremner *et al.*, 1991; Adams *et al.*, 1992). In the light of these discrepancies, a number of relevant issues need to be clarified regarding the relationship between energy status and tumour blood flow. In the present study, the key questions were as follows:

(1) Do changes in tumour blood flow necessarily lead to changes in the bioenergetic status during normoglycaemia?

(2) Is there a range of tumour blood flow rates over which the energy status is not affected by perfusion changes?

(3) Is there a critical threshold below which a positive correlation between flow and energy status may exist?

### Materials and methods

### *Animals, tumours and surgical procedures*

Sprague–Dawley rats (Charles River Wiga, Sulzfeld, Germany; body weight: 310 ± 5 g) were used for experiments. Animals were allowed access to food and water (pH 4.0) *ad libitum* prior to experiments. Experimental tumours were grown subcutaneously after injection of ascites cells of DS sarcoma into the hindfoot dorsum (Kluge *et al.*, 1992).

Once tumours reached the desired size, animals were anaesthetised with sodium pentobarbital (40 mg $kg^{-1}$ i.p., Nembutal, Ceva, Paris, France). Catheters were then surgically placed into the thoracic aorta via the left common carotid artery and into the right external jugular vein. During surgical procedures and throughout all experiments, the animals were placed supine on a heated operation pad (rectal temperature ≈37°C), such that tumour temperature was maintained within the range 34–36°C throughout all experiments. In order to monitor the mean arterial blood pressure (MABP) continuously, the arterial catheter was connected to a Statham pressure transducer (type P 23 ID, Gould, Oxnard, CA, USA). Animals breathed room air spontaneously. Oxygen ($P\text{O}_2$) and carbon dioxide ($P\text{CO}_2$) partial pressures and pH were determined in arterial blood samples (50 μl) at regular time intervals.

Relevant parameters describing tumour perfusion, bioenergetic and oxygenation status were measured either during growth of the s.c. tumours ('chronic' decrease of tumour blood flow) or following acute changes in tissue perfusion. In the latter case, TNF-α was applied in order to induce a significant flow drop within a short period of time.

### *Measurement of tumour blood flow*

Tumour blood flow (TBF) was studied using the $^{85}$Kr clearance technique. For TBF measurements the indicator (0.1 ml of a solution of $^{85}$Kr in 0.9% sodium chloride, 37 MBq $ml^{-1}$, Amersham-Buchler, Braunschweig, Germany) was applied as a bolus injection through the arterial catheter into the thoracic aorta. The registration of the washout process was performed with a Geiger-counting tube con-

Correspondence: P. Vaupel.
Received 15 June 1993; and in revised form 12 August 1993.

nected to a ratemeter (FHT 1100 FAG Kugelfischer, Erlangen, Germany). The method of evaluation of TBF was identical to that described earlier (Kluge *et al.*, 1992). Measurements were performed on tumours of varying sizes at 20 min intervals before application of TNF-α and at 30 min intervals thereafter over a total time period of 2 h post treatment. In all experiments performed in this study, animals were allowed to stabilise following the surgical procedures. Measurements commenced once constant baseline readings for MABP and flow were obtained for at least 20 min.

*Laser Doppler flowmetry*

A Periflux model PF 3 dual-channel laser Doppler flowmeter was used for this study (2 mW He-Ne laser, wavelength 632.8 nm; Perimed, Stockholm, Sweden). Laser Doppler flow (LDF) signals were continuously recorded from central locations on the tumour surface using a type PF 108 probe. The fibreoptic probe was placed above (but not in contact with) the tumour tissue under study. LDF was recorded for 10 min before i.v. administration of TNF-α or saline (control) and for 90 min thereafter (Kluge *et al.*, 1992).

*Tumour oxygen tension measurements*

Tumour oxygen tension values were determined using polarographic needle electrodes (recessed 12 μm gold in glass cathode; shaft diameter 250 μm) and $Po_2$ histography (model KIMOC-6650, Eppendorf, Hamburg, Germany) as described previously (Vaupel *et al.*, 1989*a*, 1991). Measurements were made either on tumours of varying sizes or before and 120 min after acute flow changes upon TNF-α application.

*Measurement of global concentrations of adenylate phosphates in perchloric acid extracts*

In order to obtain mean (global) levels of adenylate phosphates, the tumour-bearing hindfoot was rapidly frozen and the tumours ($n = 12$) were prepared under liquid nitrogen and stored at −80°C for further processing. In a first series of experiments, tumours of varying sizes were analysed. In another series, tumours were assayed before or 120 min after administration of TNF-α.

Each deep-frozen tumour was ground to a fine powder and freeze dried. For determination of ATP, ADP and AMP levels, aliquots of freeze-dried tissue were extracted with 0.66 M perchloric acid, centrifuged and the supernatant neutralised with 2 M potassium hydroxide. The concentrations of the adenylate phosphates were then determined using reversed-phase high-performance liquid chromatography (HPLC) techniques at 254 nm (for more details see Krüger *et al.*, 1991; Schaefer *et al.*, 1993). Concentrations were expressed as μmol per g tissue wet weight.

*Determination of microregional ATP distribution*

Before preparing the rapidly frozen tumours for HPLC analysis, approximately 30% of the tumour mass was separated, cut at −25°C in a cryostat into 5-μm sections and used for ATP bioluminescence measurements to assess the microregional ATP distribution using single-photon imaging and quantitative bioluminescence (for methodological details see Walenta *et al.*, 1992; Schaefer *et al.*, 1993). The spatial resolution gained by this method is about 50 μm, thus revealing information about the intra-tumour variability of the ATP levels in relation to histological details.

*Introduction of acute flow drops through TNF-α*

Recombinant human TNF-α (specific activity: $8.2 \times 10^6$ U per mg of protein; Knoll, Ludwigshafen, Germany) was diluted in isotonic phosphate-buffered saline solution containing 0.5% (w/w) bovine serum albumin (Sigma Chemie, Deisenhofen, Germany). TNF-α was given into the external jugular vein at a dose level of 1 mg $kg^{-1}$ over approximately 3 min. The catheter used for the i.v. route was flushed with saline thereafter. Control animals received identical fluid loads (1 ml $kg^{-1}$ phosphate-buffered saline i.v.). For further details see Kluge *et al.* (1992).

*Statistical analysis*

Results are expressed as means ± s.e. with the numbers of experiments indicated in brackets. Significance was assessed using the paired or unpaired Student's *t*-test, as appropriate. Results were considered as significant if *P*-values were less than 5% ($P<0.05$).

**Results and Discussion**

Like many other experimental tumour systems, tumour blood flow (TBF) and tissue oxygenation significantly decrease in the DS sarcoma with increasing tumour mass (see Figure 1). Starting from a mean TBF value of 0.98 ml $g^{-1}$ $min^{-1}$ in the smallest tumours investigated, flow decreased by about 50% in the larger malignancies ($2P<0.001$). This flow drop coincides with a similar decrease in the mean $Po_2$ value from 39 to 16 mmHg ($2P<0.001$).

As long as tumour masses do not exceed 1% of the body weight (i.e. biologically relevant tumour sizes), global ATP concentrations and adenylate energy charge remain almost constant. During tumour growth from 0.86 ± 0.02 to 2.15 ± 0.04 g, ATP concentrations insignificantly increased from 1.15 ± 0.10 to 1.37 ± 0.12 μmol $g^{-1}$. Similar results were obtained when the microregional ATP distribution was analysed in three tissue sections of three tumours each of three different size groups (mean tumour weights: 0.82 ± 0.08 g, 1.25 ± 0.10 g and 2.14 ± 0.12 g; see Figure 1).

Flow and $Po_2$ values in these tumour size ranges are similar to those observed in many normal tissues (Vaupel *et al.*, 1989*c*) and are seen to be accompanied by a stable energy status. Changes in TBF can influence tissue oxygenation but not ATP concentrations in this tissue. This may be explained by an intensified glycolytic rate as the oxygenation status deteriorates and/or a decreasing number of proliferating cells which compensate for the poorer oxygen supply as the tumours become larger. As long as TBF and/or $Po_2$ values do not fall below a certain 'threshold', tumour energy status can be maintained. Under these conditions, glucose has to be considered as the major energy source, which is available in sufficient amounts even under normoglycaemic conditions. Owing to the large interstitial space of those tumours [app-

**Figure 1** Tumour growth-related changes in tumour blood flow (TBF, open circles) and mean tissue oxygen tension ($Po_2$, closed circles) and mean tumour tissue ATP concentrations as determined by HPLC in perchloric acid extracts (open triangles) or quantitative bioluminescence (closed triangles). Values in parentheses indicate the numbers of tumours investigated. Values are means ± s.e. Standard errors of tumour wet weights are within the symbol sizes.

roximately 50% (v/v); Gullino et al., 1965; Vaupel & Müller-Klieser, 1983; Stubbs et al., 1992], the mean tissue glucose concentration is $>1.5\,\mu mol\,g^{-1}$ ('reservoir function' of the interstitial space). The missing decrease in high-energy phosphates despite severe restrictions in tumour blood flow during hyperglycaemia as observed in rodent tumours supports this notion (Okunieff et al., 1989b; Krüger et al., 1991; Schaefer et al., 1993). In line with these findings is a recent study of Gerweck et al. (1993) showing that energy status and oxygenation are not closely linked in the presence of glucose.

Similar observations have been described for other experimental tumour systems. In an amelanotic hamster melanoma (A-Mel-3), ATP concentrations remained constant as long as blood flow values were above $0.4\,ml\,g^{-1}\,min^{-1}$ (Walenta et al., 1992). This finding is based on pixel-to-pixel correlations between microregional ATP concentrations and flow data.

In murine FSaII tumours, median $Po_2$ values of 10–15 mmHg represent a critical threshold for energy metabolism (Vaupel et al., 1993). At higher median $Po_2$ values, ATP levels were relatively constant. On average, median oxygen tensions below 10–15 mmHg coincided with ATP depletion, intracellular acidosis, a drop in the energy charge and rising $P_i$ levels (Vaupel, 1992). These conditions were, however, only found when tumour masses were $>1.5\%$ of body weight.

Stable bioenergetic status is observed not only during 'growth-related' decreases in blood flow or tissue $Po_2$ values, but also upon acute falls in TBF following TNF-α administration. This cytokine is known to drastically reduce microcirculatory function (Kluge et al., 1992; Naredi et al., 1993). Starting from TBF values of $0.98 \pm 0.05\,ml\,g^{-1}\,min^{-1}$ (tumour wet weights: $0.85 \pm 0.05$ g), TNF-α application resulted in a 50% flow drop within 120 min (Figure 2). Similar changes were observed for the tumour $Po_2$ distribution. Despite these substantial changes, ATP levels, phosphocreatine (PCr)/$P_i$ and β-nucleoside triphosphate (β-NTP)/$P_i$ ratios remained almost unchanged*.

Here again, energy status was stable at mean flow values $\geq 0.5\,ml\,g^{-1}\,min^{-1}$, mean oxygen tensions $\geq 13$ mmHg and mean tumour tissue glucose levels $\geq 1.4\,\mu mol\,g^{-1}$ (Engel & Vaupel, 1993). From the data presented it is concluded that growth-related or acute changes in tumour perfusion are, as a rule, accompanied by parallel alterations of tissue oxygenation. In contrast, tumour energy status is stable providing flow values do not fall below a certain threshold (approximately $0.4–0.5\,ml\,g^{-1}\,min^{-1}$ in the rodent tumour systems investigated). As compensatory mechanisms, an intensified glycolysis due to the recruitment of glucose from the 'interstitial reservoir' and a decrease in the number of proliferating cells, have to be assumed.

*PCr/$P_i$ and NTP/$P_i$ ratios were obtained from $^{31}$P-NMR spectroscopy (Kluge et al., 1992) using the same experimental protocol (B. Elger et al., unpublished data).

**Figure 2** Acute effects of TNF-α ($1\,mg\,kg^{-1}$ i.v.) on tumour blood flow (TBF, open circles, $n = 28$), laser Doppler flow (LDF, closed circles, $n = 6$), mean oxygen partial pressure in tumour tissue ($Po_2$, open triangles, $n = 12$), tumour tissue ATP concentrations (closed triangles, $n = 12$) and $^{31}$P-magnetic resonance spectroscopy-derived PCr/$P_i$ (closed squares), and β-NTP/$P_i$ ratios (open squares, $n = 5$). Values are means ± s.e.

DS sarcoma was kindly provided by Dr H. Löhrke from the German Cancer Research Centre in Heidelberg.

This work was supported by grants from the Deutsche Krebshilfe (Grant M 40/91/Va 1) and from the Vinzenz von Paul-Foundation, Basle, Switzerland (Grant 5.1).

Abbreviations: ADP, adenosine diphosphate; AMP, adenosine monophosphate; ATP, adenosine triphosphate; HPLC, high-performance liquid chromatography; LDF, laser Doppler flow; MABP, mean arterial blood pressure; NTP, β-nucleoside triphosphate; PCr, phosphocreatine, $P_i$, inorganic phosphate; $Po_2$, oxygen partial pressure; TBF, tumour blood flow; TNF-α, tumour necrosis factor α.

### References

ADAMS, G.E., BREMNER, J.C.M., COUNSELL, C.J.R., STRATFORD, I.J., THOMAS, C. & WOOD, P.J. (1992). Magnetic resonance spectroscopy studies on experimental murine and human tumours: comparison of changes in phosphorus metabolism with induced changes in vascular volume. *Int. J. Radiat. Oncol. Biol. Phys.*, **22**, 467–471.

BREMNER, J.C.M., COUNSELL, C.J.R., ADAMS, G.E., STRATFORD, I.J., WOOD, P.J., DUNN, J.F. & RADDA, G.K. (1991). *In vivo* $^{31}$P nuclear magnetic resonance spectroscopy of experimental murine tumours and human tumour xenografts: effects of blood flow modification. *Br. J. Cancer*, **64**, 862–866.

BREMNER, J.C.M., BRADLEY, J.K., COUNSELL, C.J.R. & ADAMS, G.E. (1993). Changes in $^{31}$P-metabolism and blood flow after photodynamic therapy (PDT): a comparison between a murine sarcoma (RIF-1) and a human xenografted tumour (HT29). Abstract P-08-1. *41st Annual Meeting of the Radiation Research Society, Dallas, TX, March 20–25.*

CONSTANTINIDIS, I., BRAUNSCHWEIGER, P.G., WEHRLE, J.P., KUMAR, N., JOHNSON, C.S., FURMANSKI, P. & GLICKSON, J.D. (1989). $^{31}$P-nuclear magnetic resonance studies of the effect of recombinant human interleukin 1α on the bioenergetics of RIF-1 tumors. *Cancer Res.*, **49**, 6379–6382.

ENGEL, T. & VAUPEL, P. (1993). Acute effects of tumor necrosis factor-α or lymphotoxin on oxygenation and bioenergetic status of experimental tumors. *Adv. Exp. Med. Biol.* (in press).

EVELHOCH, J.L., SAPARETO, S.A., NUSSBAUM, G.H. & ACKERMAN, J.H. (1986). Correlations between $^{31}$P NMR spectroscopy and $^{15}$O perfusion measurements in the RIF-1 murine tumor *in vivo*. *Radiat. Res.*, **106**, 122–131.

GERWECK, L.E., SENEVIRATNE, T. & GERWECK, K.K. (1993). Energy status and radiobiological hypoxia at specified oxygen concentrations. *Radiat. Res.*, **135**, 69–74.

GULLINO, P.M., GRANTHAM, F.H. & SMITH, S.H. (1965). The interstitial water space of tumors. *Cancer Res.*, **25**, 727–731.

KLUGE, M., ELGER, B., ENGEL, T., SCHAEFER, C., SEEGA, J. & VAUPEL, P. (1992). Acute effects of tumor necrosis factor α or lymphotoxin on global blood flow, laser Doppler flux, and bioenergetic status of subcutaneous rodent tumors. *Cancer Res.*, **52**, 2167–2173.

KRÜGER, W., MAYER, W.-K., SCHAEFER, C., STOHRER, M. & VAUPEL, P. (1991). Acute changes of systemic parameters in tumour-bearing rats, and of tumour glucose, lactate, and ATP levels upon local hyperthermia and/or hyperglycaemia. *J. Cancer Res. Clin. Oncol.*, **117**, 409–415.

LILLY, M.B., KATHOLI, C.R. & NG, T.C. (1985). Direct relationship between high-energy phosphate content and blood flow in thermally treated murine tumors. *J. Natl Cancer Inst.*, **75**, 885–889.

MAYER, W.-K., STOHRER, M., KRÜGER, W. & VAUPEL, P. (1992). Laser Doppler flux and tissue oxygenation of experimental tumours upon local hyperthermia and/or hyperglycaemia. *J. Cancer Res. Clin. Oncol.*, **118**, 523–528.

NAREDI, P.L.J., LINDNER, P.G., HOLMBERG, S.B., STENRAM, U., PETERSON, A. & HAFSTRÖM, L.R. (1993). The effects of tumour necrosis factor alpha on the vascular bed and blood flow in an experimental rat hepatoma. *Int. J. Cancer*, **54**, 645–649.

OKUNIEFF, P., WALSH, C.S., VAUPEL, P., KALLINOWSKI, F., HITZIG, B.M., NEURINGER, L.J. & SUIT, H.D. (1989*a*). Effects of hydralazine on *in vivo* tumor energy metabolism, hematopoietic radiation sensitivity, and cardiovascular parameters. *Int. J. Radiat. Oncol. Biol. Phys.*, **16**, 1145–1148.

OKUNIEFF, P., VAUPEL, P., SEDLACEK, R. & NEURINGER, L.J. (1989*b*). Evaluation of tumor energy metabolism and microvascular blood flow after glucose or mannitol administration using $^{31}$P nuclear magnetic resonance spectroscopy and laser Doppler flowmetry. *Int. J. Radiat. Oncol. Biol. Phys.*, **16**, 1493–1500.

SCHAEFER, C., MAYER, W.-K., KRÜGER, W. & VAUPEL, P. (1993). Microregional distributions of glucose, lactate, ATP and tissue pH in experimental tumours upon local hyperthermia and/or hyperglycaemia. *J. Cancer Res. Clin. Oncol.*, **119**, 599–608.

SIJENS, P.E., BOVEE, W.M.M.J., SEIJKENS, D., KOOLE, P., LOS, G. & VAN RIJSSEL, R.H. (1987). Murine mammary tumor response to hyperthermia and radiotherapy evaluated by *in vivo* $^{31}$P-nuclear magnetic resonance spectroscopy. *Cancer Res.*, **47**, 6467–6473.

STEEN, R.G. & GRAHAM, M.M. (1991). $^{31}$P magnetic resonance spectroscopy is sensitive to tumor hypoxia: perfusion and oxygenation of rat 9L gliosarcoma after treatment with BCNU. *NMR Biomed.*, **4**, 117–124.

STUBBS, M., BHUJWALLA, Z.M., TOZER, G.M., RODRIGUES, L.M., MAXWELL, R.J., MORGAN, R., HOWE, F.A. & GRIFFITHS, J.R. (1992). An assessment of $^{31}$P MRS as a method of measuring pH in rat tumours. *NMR Biomed.*, **5**, 351–359.

TOZER, G., SUIT, H.D., BARLAI-KOVACH, M., BRUNENGRABER, H. & BIAGLOW, J. (1987). Energy metabolism and blood perfusion in a mouse mammary adenocarcinoma during growth and following X irradiation. *Radiat. Res.*, **109**, 275–293.

TOZER, G.M., BHUJWALLA, Z.M., GRIFFITHS, J.R. & MAXWELL, R.J. (1989). Phosphorus-31 magnetic resonance spectroscopy and blood perfusion of the RIF-1 tumor following X-irradiation. *Int. J. Radiat. Oncol. Biol. Phys.*, **16**, 155–164.

TOZER, G.M., MAXWELL, R.J., GRIFFITHS, J.R. & PHAM, P. (1990). Modification of the $^{31}$P magnetic resonance spectra of a rat tumour using vasodilators and its relationship to hypotension. *Br. J. Cancer*, **62**, 553–560.

VAUPEL, P. (1992). Physiological properties of malignant tumours. *NMR Biomed.*, **5**, 220–225.

VAUPEL, P. & MÜLLER-KLIESER, W. (1983). Interstitieller Raum und Mikromilieu in malignen Tumoren. *Mikrozirk. Forsch. Klin.*, **2**, 78–90.

VAUPEL, P., OKUNIEFF, P., KALLINOWSKI, F. & NEURINGER, L.J. (1989*a*). Correlations between $^{31}$P-NMR spectroscopy and tissue $O_2$ tension measurements in a murine fibrosarcoma. *Radiat. Res.*, **120**, 477–493.

VAUPEL, P., OKUNIEFF, P. & NEURINGER, L.J. (1989*b*). Blood flow, tissue oxygenation, pH distribution, and energy metabolism of murine mammary adenocarcinomas during growth. *Adv. Exp. Med. Biol.*, **248**, 835–845.

VAUPEL, P., KALLINOWSKI, F. & OKUNIEFF, P. (1989*c*). Blood flow, oxygen and nutrient supply, and metabolic microenvironment of human tumors: a review. *Cancer Res.*, **49**, 6449–6465.

VAUPEL, P., SCHLENGER, K., KNOOP, C. & HOECKEL, M. (1991). Oxygenation of human tumors: Evaluation of tissue oxygen distribution in breast cancers by computerized $O_2$ tension measurements. *Cancer Res.*, **51**, 3316–3322.

VAUPEL, P., SCHAEFER, C. & OKUNIEFF, P. (1993). Intracellular acidosis in murine fibrosarcomas coincides with ATP depletion, hypoxia, and high levels of lactate and total $P_i$. *NMR Biomed.* (in press).

WALENTA, S., DELLIAN, M., GOETZ, A.E., KUHNLE, G.E.H. & MUELLER-KLIESER, W. (1992). Pixel-to-pixel correlation between images of absolute ATP concentrations and blood flow in tumours. *Br. J. Cancer*, **66**, 1099–1102.